# Immunogenicity and Safety of COVID-19 Vaccines in Patients Receiving Renal Replacement Therapy: A Systematic Review and Meta-Analysis

**DOI:** 10.3389/fmed.2022.827859

**Published:** 2022-03-09

**Authors:** Becky Mingyao Ma, Anthony Raymond Tam, Kam Wa Chan, Maggie Kam Man Ma, Ivan Fan Ngai Hung, Desmond Yat Hin Yap, Tak Mao Chan

**Affiliations:** ^1^Division of Nephrology, Department of Medicine, Queen Mary Hospital, The University of Hong Kong, Pokfulam, Hong Kong SAR, China; ^2^Division of Infectious Diseases, Department of Medicine, Queen Mary Hospital, The University of Hong Kong, Pokfulam, Hong Kong SAR, China

**Keywords:** COVID-19, vaccine, immunogenicity, safety, chronic kidney disease

## Abstract

**Background:**

Systematic data on the efficacy and safety of COVID-19 vaccine in patients on renal replacement therapy (RRT) remains limited. We conducted a meta-analysis on the efficacy and safety of COVID-19 vaccine in patients on RRT.

**Methods:**

Eligible studies were identified by systematic literature search in four electronic databases. Twenty-seven studies (4,264 patients) were included for meta-analysis. 99% patients received mRNA vaccine.

**Results:**

Patients on RRT showed inferior seropositivity after two-dosed COVID-19 vaccine, 44% lower than the general population. Kidney transplant recipients (KTRs) had significantly lower seropositivity than patients on haemodialysis (HD) or peritoneal dialysis (PD) (26.1 vs. 84.3% and 92.4% respectively, *p* < 0.001 for both). Compared with healthy controls, KTRs, HD and PD patients were 80% (95% CI: 62–99%), 18% (95% CI: 9–27%) and 11% (95% CI: 1–21%) less likely to develop antibodies after vaccination (*p* < 0.001, <0.001 and 0.39 respectively). In KTRs, every 1% increase in using mycophenolate was associated with 0.92% reduction in seropositivity (95% CI: −1.68, −0.17, *p* = 0.021) at population level. The overall adverse event rate attributed to vaccination was 2.1%. Most events were mild.

**Conclusion:**

Patients on RRT, particularly KTRs, had significantly reduced antibody response after two-dosed COVID-19 vaccination. Vaccination is generally well tolerated.

**Systematic Review Registration:**

https://www.crd.york.ac.uk/prospero/, identifier: CRD42021261879.

## Introduction

Coronavirus disease 2019 (COVID-19), caused by severe acute respiratory syndrome coronavirus 2 (SARS-CoV-2), continues to be a global public health threat causing significant patient morbidity and mortality. Various treatments have been proposed to manage COVID-19, largely based on drug repurposing of existing anti-virals and to a lesser extent novel therapeutic compounds (1). In this context, clinical trials on anti-virals for hospitalized patients with severe COVID-19 showed mixed results. Remdesivir is the only drug that has been approved by the Food and Drug Administration for the treatment of COVID-19 (2). Prevention of COVID-19 through effective immunization therefore remains an important armamentarium in combating the pandemic.

The speed of COVID-19 vaccine development was unprecedented. As of November 5, 2021, 329 vaccines using various platforms were registered into clinical and preclinical trials (3). These platforms ranged from time-honored methods using inactivated virus, to novel approaches employing nucleic acids or viral vectors (4). Messenger ribonucleic acid (mRNA) vaccine platform is a new technology mimicking native nucleic acid translation to produce target proteins that are presented to lymphocytes for generation of immunological memory. Phase 3 clinical trials of two mRNA vaccines (BNT126b2 and mRNA-1273) reported vaccine efficacies of 95% and 94.1% respectively in the general population (5, 6). The mRNA sequences contained in these vaccines encode the spike (S) protein of SARS-CoV-2, which interacts with angiotensin-converting enzyme-2 (ACE-2) on surfaces of human cells mediating cellular entry. Cohorts of healthy adults showed immunogenicity of 100% after the second dose of mRNA vaccination (7). The ChAdOx1 nCoV-19 vaccine, a viral vector vaccine, has been found to be efficacious against symptomatic COVID-19 with a reported immunogenicity of over 99% and vaccine efficacy of 70.4% after two standard doses (8). Ad26.COV2.S, an adenovirus vector vaccine, requires only one dose to generate antibodies stably in 96% of participants at days 56 and 71 and has a protection rate against SARS-CoV-2-related hospitalization up to 68% (9). For the inactivated viral vaccine (CoronaVac), seroconversion rates were 86.7 and 70.4% in healthy subjects of age 18–59 and ≥ 60 respectively (10).

Patients with chronic kidney disease (CKD) are at increased risk of COVID-19 infection, severe complications and death (11), and thus effective and safe vaccination is particularly important for these patients. However, it is well recognized that CKD patients have reduced vaccine response due to altered innate, cellular and humoral immunity. The immunogenicity of vaccines decreases with severity of renal impairment, in which patients on dialysis or kidney transplantation have significantly worse immune response to vaccination compared with the general population and non-dialysis CKD patients. Generally, KTRs receive immunosuppressive regimen comprising corticosteroids, calcineurin inhibitors (CNIs), mammalian target of rapamycin (mTOR) inhibitors or anti-metabolites (e.g., mycophenolate), and these drugs can significantly attenuate vaccine's immunogenicity. While there is emerging data on the immunogenicity and safety of COVID-19 vaccine in CKD patients (12–38), these studies showed considerable heterogeneities in patient characteristics, type of vaccines administered, assays used for measuring antibodies and healthcare settings, which rendered comparison of results and drawing of conclusions difficult. Based on these knowledge gaps, we conducted a meta-analysis to investigate the overall immunogenicity and safety of COVID-19 vaccines in patients on renal replacement therapy (RRT) and also identify factors that affect vaccine immunogenicity. The results from this meta-analysis will provide useful information regarding COVID-19 vaccines in highly vulnerable CKD patients, namely those on dialysis or kidney transplantation.

## Materials and Methods

### Search Strategy

The study was registered under the international prospective register of systematic reviews (PROSPERO, registration ID: CRD42021261879) and followed the standards set forth by the PRISMA (Preferred Reporting Items for Systematic Reviews and Meta-Analyses) statement (39). We searched systematically in PubMed, Cochrane Library, Medline and EMBase to identify all published and pre-publication studies on COVID-19 vaccines in CKD patients up to 30^th^ June 2021 (keywords used and search strategy were listed in Supplementary Material S1). The search is limited to English language papers.

### Study Selection

Clinical trials and cohort studies of adults (aged 18 years or older) with CKD on HD, peritoneal dialysis (PD) or KTRs without a prior history of COVID-19, who have received two doses of COVID-19 vaccines and have antibody titres tested, were included. Reviews, case reports, commentaries, conference abstracts and animal studies were excluded. Studies were excluded if there was an overlap in subjects with another included study, hence study subjects were only included once in any given analysis.

### Data Extraction and Quality Assessment

Data was extracted and reviewed by three investigators (BMM, ART and DY) independently, and disagreements were resolved by their consensus or consultation with the fourth author. Data on the journal, publication date, first author, study design, sample size, patient demographics (including age, sex, modality of renal replacement therapy, vintage of dialysis or transplantation, immunosuppressive regimen, history of diabetes mellitus), number of healthy controls, type and regimen of COVID-19 vaccination, antibody response after second dose of vaccination and adverse reactions were collected. Risk of bias was assessed using the National Institute of Health Study Quality Assessment Tool (Supplementary Material S2) (40).

### Outcomes

Main outcomes of our meta-analysis were the immunogenicity and safety of COVID-19 vaccine in patients on RRT. The immunogenicity was evaluated by antibody response to the receptor-binding domain of the spike protein and antibodies to SARS-CoV-2 using commercially available assays and was expressed as the pooled percentage of seropositivity. The assays used in all included studies and the respective cut-off values for seropositivity were listed in Supplementary Material S3. Safety was evaluated by occurrence of any local or systemic adverse reaction.

### Statistical Analysis

Continuous variables were expressed as mean ± SD. The post-vaccination antibody levels in patients on RRT and healthy controls were pooled, stratified across studies, and analyzed using random-effect models with inverse variance weighting. The magnitude of heterogeneity was estimated using the I^2^ statistic, an estimate of the proportion of the total observed variance that is attributed to between-study variance. The pooled effects of antibody levels were presented as weighted risk difference with corresponding 95% CI. Univariate meta-regression was used to investigate differences for categorical variables. All statistical analyses were performed using STATA version 17.0 (College Station, Texas, USA), and p-values of <0.05 were considered statistically significant.

## Results

### Data Source and Patient Characteristics

**Figure 1:**
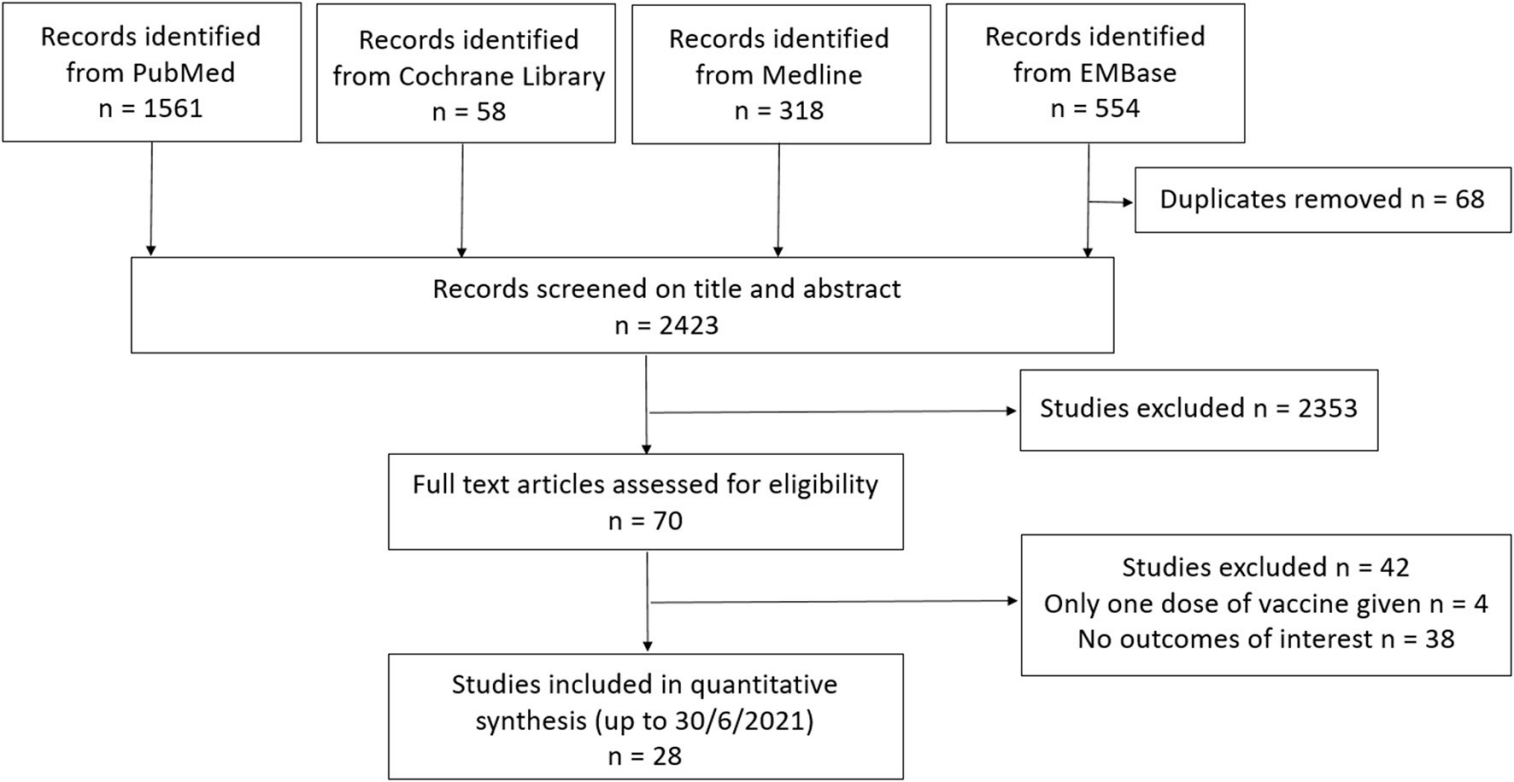
Flowchart of study selection.

A search of PubMed, Cochrane Library, Medline and EMBase databases using pre-determined search criteria generated 2491 studies initially (Figure 1). Duplicates, *in vitro* studies, studies using animal models or conducted in pediatric population were excluded. Seventy studies were retrieved for more detailed evaluation by full-text screening and 43 studies were excluded since they did not report vaccine response or only reported serologic response after first dose of vaccine. Twenty-seven studies from 8 countries were included in the final analysis (Supplementary Material S4) (12–38). Eleven studies had healthy adults as control group. A total of 4,264 patients (1928 KTRs, 2215 receiving chronic HD and 121 receiving PD) with no prior history of COVID-19 were included for meta-analysis. The mean age of patients on RRT and healthy controls of included studies were 62.5 and 49.7 years respectively. Majority of patients received mRNA platform vaccines (99%) and the rest (1%) received viral vector platform vaccine. Among those receiving mRNA platform vaccines, 74.9% had BNT162b2 and 25.1% had mRNA-1273. All studies adopted a two-dose vaccination protocol. The antibody response to the receptor-binding domain of the spike protein of SARS-CoV-2 was measured using seven different assays (Supplementary Material S3), in which up to 50% of the studies used the ABBOTT or LIAISON assays for the measurement of anti-SARS-CoV-2 antibody. Blood was sampled 7–42 days after the second dose vaccine.

### Antibody Response in Patients Receiving Different RRT Modalities

**Figure 2:**
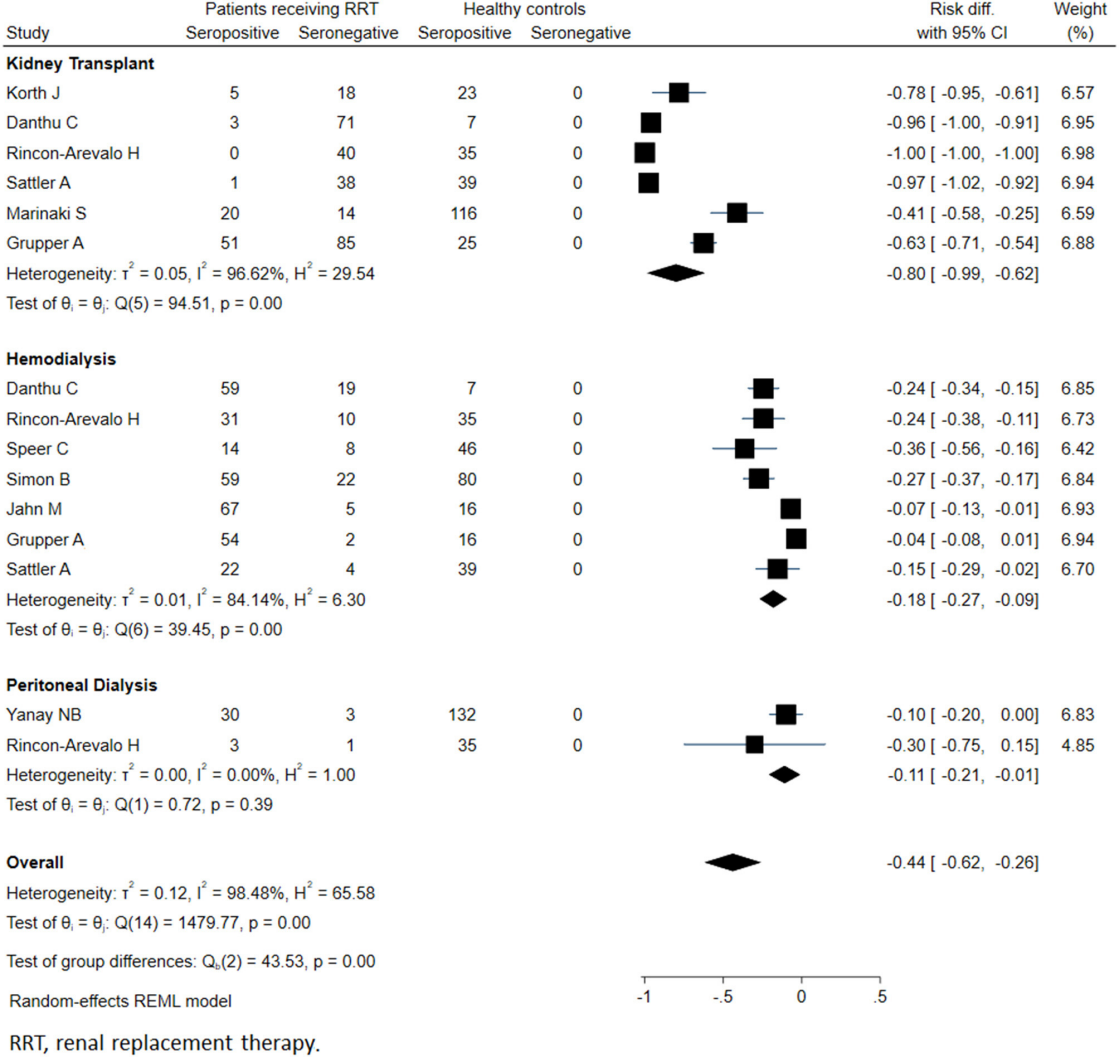
Immunogenicity after two doses of COVID-19 vaccines in patients receiving renal replacement therapy and healthy subjects.

Meta-analysis of twenty-seven studies (*n* = 4264) showed that the overall seropositivity rate of anti-SARS-CoV-2 was 64% in patients on RRT. Compared to healthy controls, patients on RRT showed a 44% absolute reduction in the seropositivity rates (95% CI: −26%, −62%; *p* < 0.001) (Figure 2).

**Figure 3:**
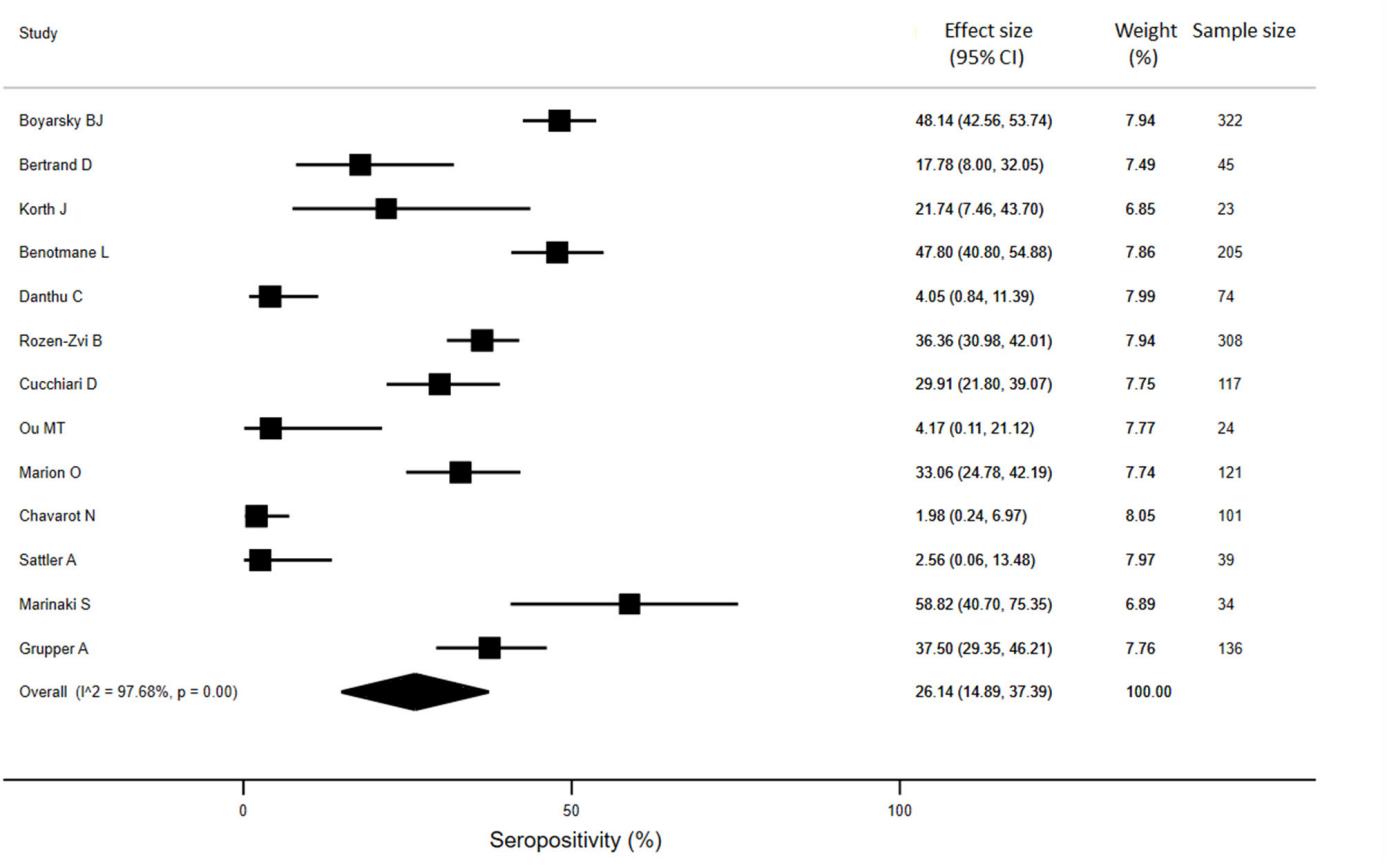
Immunogenicity after two doses of COVID-19 vaccines in kidney transplant recipients.

**Figure 4:**
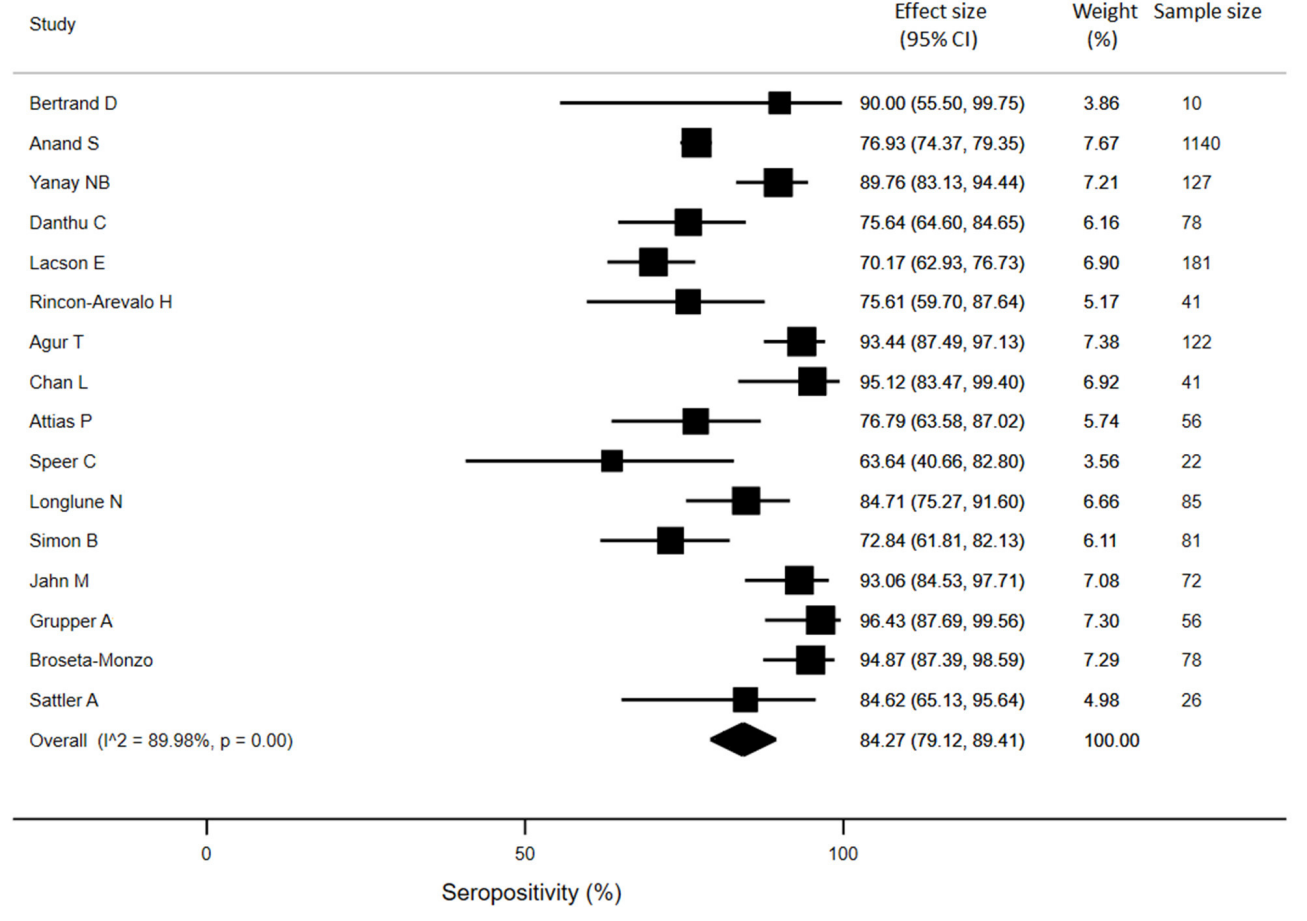
Immunogenicity after two doses of COVID-19 vaccines in patients receiving chronic hemodialysis.

**Figure 5:**
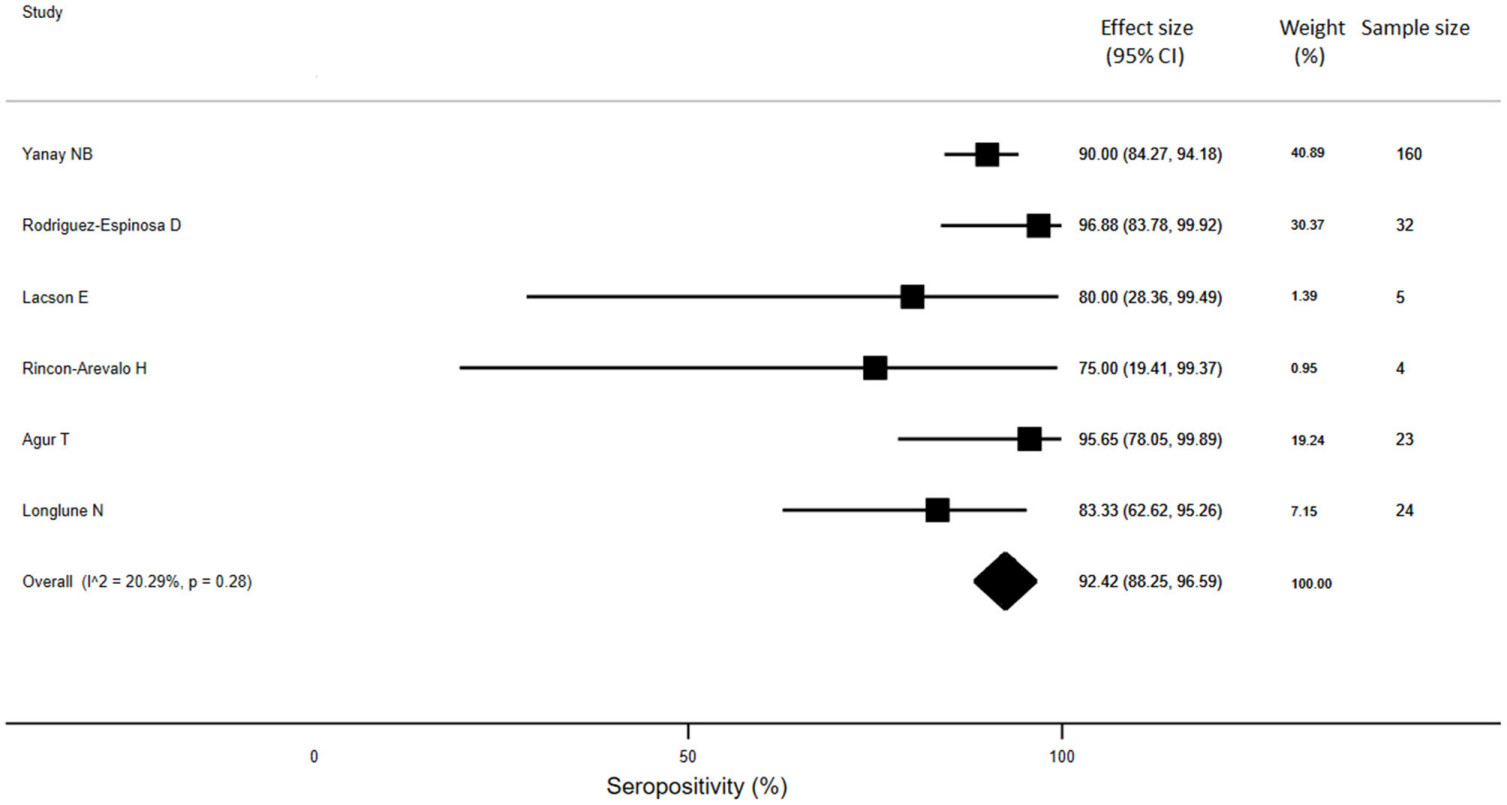
Immunogenicity after two doses of COVID-19 vaccines in patients receiving peritoneal dialysis.

For KTRs, a meta-analysis of 13 studies (*n* = 1888) revealed that only 26% of patients developed anti-SARS-CoV-2 antibodies (95% CI: 14.9%, 37.4%) (Figure 3). Compared with healthy individuals, KTRs were 80% less likely to be seropositive for anti-SARS-CoV-2 (95% CI: −62%, −99%; *p* < 0.001). Regarding patients on chronic HD, meta-analysis of 16 studies (*n* = 2175) showed a seropositivity rate of 84.3% (95% CI: 79.1%, 89.4%) (Figure 4). Compared to general population, patients receiving HD were 18% less likely to achieve seropositivity (95% CI: −9%, −27%; *p* < 0.001). For patients on PD, meta-analysis of six studies (*n* = 121) showed a seropositivity rate of 92.4% (95% CI: 88.3%, 96.6%) (Figure 5). Compared to healthy subjects, patients on PD were 11% less likely to become seropositive though not reaching statistical significance (95% CI: −1%, −21%; *p* = 0.39).

The mean titres of antibodies after second dose in patients receiving different modalities of RRT were summarized in Supplementary Material S3. Data from 4 studies showed that patients on chronic HD developed higher levels of anti-SARS-CoV-2 antibodies compared to KTRs though the difference did not reach statistical significance [LIAISON assay – 467 ± 434 AU/mL vs. 32 ± 77 AU/mL, *p* = 0.053; ABBOTT assay – 1907 ± 2741 AU/mL vs. 65 ± 233 AU/mL, *p* = 0.189]. Pooled data from 5 studies showed that patients on PD developed comparable levels of anti-SARS-CoV-2 antibodies with patients receiving chronic HD [IgG index value (SIEMENS assay): 16 ± 8 vs. 14 ± 15 in PD and HD respectively, *p* = 0.678].

### Factors That Affect the Immunogenicity in Patients Receiving RRT

We investigated the impact of different immunosuppressants and medical co-morbidities on the immunogenicity of COVID-19 vaccine in KTRs. Our meta-regression demonstrated that for every 1% increase in using mycophenolate mofetil (MMF) among KTRs, there was a 0.92% decrease in number of patients who developed anti-SARS-CoV2 antibodies (95% CI −1.68, −0.17, *p* = 0.021). The other immunosuppressants including corticosteroids, calcineurin inhibitors, mTOR inhibitors had no statistically significant impact on seropositivity (*p* > 0.05, for all). Age, presence of diabetes mellitus (DM) and duration of kidney transplantation also showed no statistically significant correlation with seropositivity (*p*>0.05, for all).

We also analyzed the effect of different patient characteristics on the development of anti-SARS-CoV-2 antibodies in patients receiving long-term dialysis. Age, presence of DM and dialysis vintage showed no statistically significant correlation with anti-SARS-CoV-2 seropositivity among patients receiving long-term dialysis (*p*>0.05).

### Adverse Events

Only five studies (three in dialysis populations and two in KTRs) reported local or systemic adverse events after COVID-19 vaccination (Supplementary Material S4). 17 patients developed adverse events, giving an overall adverse event rate of 2.1%. Agur et al. reported one case of syncope in a HD patient 1 day post-vaccination and another case of pericarditis 2 days after the second dose of vaccine in a PD patient (31). Simon et al. demonstrated that healthy controls experienced more local (pain, redness and induration) and systemic adverse events (fatigue, headache, joint pain, fever and gastrointestinal upset) compared with the HD cohort (*p* < 0.001) (36). None of the reported adverse events resulted in emergency department attendance or hospitalization in both HD cohort and healthy controls. There was no adverse event reported after receiving 2 doses of COVID-19 vaccines in the two studies on KTRs.

## Discussion

Patients on RRT are at increased risk for severe disease manifestations, hospitalization, intensive care unit admissions and mortality when infected with SARS-CoV-2. COVID-19 vaccination has proven efficacy in alleviating disease severity and reducing mortality in the general population (5), and hence effective immunization holds promise to minimize adverse outcomes in dialysis and kidney transplant patients infected by SARS-CoV-2. However, it is well recognized that patients on RRT showed impaired immune response to vaccination (12), and studies on COVID-19 vaccines in CKD patients showed substantial heterogeneities in patient characteristics, type of vaccines administered, assays used for measuring antibodies and healthcare settings. Systematic review and analysis of efficacy and safety data of COVID-19 vaccine in patients on RRT are therefore eagerly awaited.

Our current results demonstrated that patients on RRT showed a 44% absolute reduction in the seropositivity rate of antibodies after two doses of COVID-19 vaccines compared with the general population, and the reduced seropositivity rate is largely driven by poor immunogenicity in KTRs. In this context, KTRs showed significantly lower titres and seropositivity rates of anti-SARS-CoV-2 antibodies after 2 doses of COVID-19 vaccine compared to both dialysis patients and the general population. In this meta-analysis, only 26% KTRs developed antibodies after two doses of COVID-19 vaccines, while the rest remained seronegative despite completion of immunization. This is in line with poor immunological response to HBV and influenza vaccine observed in KTRs. While CKD patients generally exhibit poor immune responses to immunization, such low immunogenicity of COVID-19 vaccine in KTRs is primarily contributed by the use of immunosuppressants. In general, KTRs receive triple immunosuppression that include corticosteroids, CNIs in combination with anti-metabolites (e.g., mycophenolic acid analogs) or mTOR inhibitors. Corticosteroids have anti-inflammatory effects via action on glucocorticoid receptors on monocytes or macrophages. CNIs bind to cyclophilin and inhibit calcineurin phosphatase in T cells, thereby specifically hampering T cell activation. Mycophenolic acid inhibits the synthesis of guanosine monophosphate nucleotides and exerts potent suppressive effects on B cell proliferation and differentiation as well as T helper cell functions – which are all key processes in generating antibodies and T cell immunity after vaccination (41). mTOR inhibitors downregulate the proliferation of T and B lymphocytes. Here we demonstrated that the use of MMF had significant impact on the development of anti-SARS-CoV2 antibodies among KTRs after COVID-19 vaccination, in which every 1% increase in using MMF was accompanied by a 0.92% decrease in patients who developed antibodies after two doses of COVID-19 vaccine. Previous studies have shown that MMF is associated with blunted immune response after influenza and pneumococcal vaccination (42). In this meta-analysis, we did not have information regarding the exposure of MMF (e.g., dose of MMF and blood mycophenolic acid levels) which may further generate important data to guide the use of COVID-19 vaccine in KTRs receiving MMF. The lack of association between corticosteroids and COVID-19 immunogenicity in our meta-analysis may be related to the relatively low dose of corticosteroids used in stable KTRs. We also did not find a significant correlation between CNIs and COVID-19 vaccine response, which again may be due to the relatively low exposure to CNIs in stable KTRs and that CNIs exert more specific inhibitory actions on T lymphocytes. mTOR inhibitors also did not show an impact on the immunogenicity of COVID-19 vaccine in KTRs, which might be explained by the small number of patients on mTOR inhibitors in the studies included for meta-analysis. Our present observation of poor COVID-19 vaccine response in KTRs supported the need for third dose of COVID-19 vaccine in these susceptible individuals. Indeed, recent data suggested that a third dose COVID-19 vaccine could increase the seropositivity rate from 40 to 68% in KTRs with no serious adverse events reported (43). Heterologous vaccination strategy with a third dose being a vector-based vaccine demonstrated comparable immunogenicity and safety to that of mRNA vaccine among KTRs who remained seronegative after 2 doses of mRNA vaccine (44). Furthermore, based on the experience of using intradermal route or adjuvants for HBV and influenza vaccines in immunocompromised hosts (45), it is also worthwhile to investigate whether these approaches may potentially enhance the immunogenicity and clinical efficacy of COVID-19 vaccines in KTRs.

Compared to KTRs, the immunogenicity of COVID-19 vaccine are affected to a lesser extent in dialysis patients. Our present findings suggested that HD patients were only 18% less likely to develop protective antibodies after two doses of COVID-19 vaccine than the general population. The antibody response after two doses of COVID-vaccine in PD patients were also comparable to healthy subjects though the lack of difference might be due to the small number of PD patients included in the meta-analysis. Previous meta-analysis of 61 studies (*n* = 6628) examining immunogenicity of hepatitis B vaccines in dialysis population showed a reduced sero-response rate of 69% compared to over 95% in healthy adults (46). Similar findings were also observed in dialysis patients receiving seasonal influenza vaccine compared to healthy controls (seroconversion rates: 43 vs. 73% for H1N1 strain and 36 vs. 62% for B strain) (47). Dialysis patients show various immunological dysfunctions that contribute to the reduced vaccine response. These immunological abnormalities include impaired endocytosis and maturation of monocytes, reduced expression of toll-like receptor and co-stimulation molecules on immune-reactive cells, impaired T cell activation and proliferation related to decreased autocrine T cell cytokines, and also diminished number of naïve and memory B cells (48, 49). Our results are reassuring as dialysis patients show respectable rates of antibodies after two doses of COVID-19. The durability of protective antibodies and also the clinical efficacy to prevent severe COVID-19 and mortality in dialysis patients, however, warrants further investigation.

One major hurdle to universal COVID-19 immunization is public anxiety on vaccine safety. In this context, patients on RRT often express much worries and concerns as they have high burdens of medical comorbidities such as DM, hypertension and cardiovascular complications. Indeed, data regarding the safety of COVID-19 vaccine in CKD population remains limited. Our current meta-analysis suggested that COVID-19 vaccine was generally safe in patients on RRT, and adverse events especially serious ones are relatively uncommon. Such observation was in line with that in the general population, in which the adverse effects of COVID-19 vaccine were mostly mild to moderate in severity and transient (50).

There are several limitations of our study. Firstly, studies included in this meta-analysis largely focused on the seropositivity and levels of antibodies in patients on RRT, and other important data on cellular immunity and clinical efficacy were often not reported. Secondly, our study did not address the durability of antibodies as it is recognized that CKD patients often show a more rapid decay in the antibody titres after vaccination (50). Thirdly, emerging data demonstrated that KTRs developed a more delayed humoral response after natural infection compared to the general population. Immunogenicity data beyond the immediate post-vaccination period are important in characterizing the full immune response profile in KTRs (51). Fourthly, most of the immunogenicity data comes from mRNA vaccines while literature on viral-vector based vaccines remain limited. Furthermore, only a small number of studies have clearly reported the adverse events in patients on RRT. Lastly, studies included in this meta-analysis are predominantly from Caucasian populations, which limits the generalizability of these data in patients of other ethnic/racial backgrounds. Notwithstanding, this study is the first comprehensive systematic review of studies on the immunogenicity and safety of COVID-19 vaccine in patients on RRT and thus provides useful information on the use of these vaccines in dialysis and kidney transplant patients.

## Conclusion

COVID-19 vaccine shows significantly reduced antibody response in patients on RRT especially among KTRs, and is generally well tolerated. The results support an additional dose of COVID-19 vaccine in KTRs and call for novel strategies to enhance immunogenicity in these susceptible patients.

## Data Availability Statement

The original contributions presented in the study are included in the article/Supplementary Material, further inquiries can be directed to the corresponding author.

## Author Contributions

BM, DY, MM, and TC conceptualized the study. BM and AT were responsible for data curation and formal analysis. KC were responsible for methodology. BM wrote the original draft. All authors reviewed and edited the manuscript. All authors contributed to the article and approved the submitted version.

## Conflict of Interest

DY received research donations from the Wai Im Charitable Foundation and the Chan Sui Kau Family Benefits and Charitable Foundation. DY and TM received research funding support from the Mr and Mrs Tam Wing Fun Edmund Renal Research Fund. TM also received research funding support from the Wai Hung Charitable Foundation and Mr. S. Ho. The donors have no role in the conduction of study. The remaining authors declare that the research was conducted in the absence of any commercial or financial relationships that could be construed as a potential conflict of interest.

## Publisher's Note

All claims expressed in this article are solely those of the authors and do not necessarily represent those of their affiliated organizations, or those of the publisher, the editors and the reviewers. Any product that may be evaluated in this article, or claim that may be made by its manufacturer, is not guaranteed or endorsed by the publisher.

## Abbreviations

ACE-2: Angiotensin converting enzyme 2
CKD: Chronic kidney disease
CNI: Calcineurin inhibitor
COVID-19: Coronavirus disease 2019
DM: Diabetes mellitus
HD: Haemodialysis
KTR: Kidney transplant recipient
MMF: Mycophenolate mofetil
mRNA: messenger ribonucleic acid
PD: Peritoneal dialysis
RRT: Renal replacement therapy
SARS-CoV-2: Severe acute respiratory syndrome coronavirus 2
SD: Standard deviation.
