# Supplementary material for: Immunogenicity and Safety of COVID-19 Vaccines in Patients Receiving Renal Replacement Therapy: A Systematic Review and Meta-Analysis
Source: Front Med (Lausanne). 2022 Mar 9;9:827859. doi: 10.3389/fmed.2022.827859 (PMC8959490; doi:10.3389/fmed.2022.827859)
Supplement: Supplementary file 1 [file Table_1.DOCX]

**Supplementary Material**

**S1. Search strategy of the systematic review/meta-analysis**

**Electronic database**

**I PubMed (as of 2021.6.30)**

((coronavir OR COVID OR nCoV OR SARS) AND (vaccine OR vaccination OR immunization OR immunisation)) AND (kidney OR renal OR nephro OR CKD OR ESRF OR ESKD OR transplant OR dialysis OR haemodialysis OR hemodialysis)

**PubMed total: 1561**

**II Cochrane Library (as of 2021.6.30)**

#1 coronavirus

#2 COVID

#3 nCoV

#4 coronavir

#5 SARS

#6 #1 OR #2 OR #3 OR #4 OR #5

#7 Kidney

#8 Renal

#9 CKD

#10 ESKD

#11 Dialysis

#12 Hemodialysis

#13 Transplant

#14 ESRD

#15 #7 OR #8 PR #9 OR #10 OR #11 OR #12 OR #13 OR #14

#16 Vaccine

#17 Vaccination

#18 Immunization

#19 Immunisation

#20 #16 OR #17 OR #18 OR #19

#21 #6 AND #15 AND #20

**Cochrane Library total: 58**

**III Medline via OVID (as of 2021.6.30)**

((coronavir OR COVID OR nCoV OR SARS) AND (vaccine OR vaccination OR immunization OR immunisation)) AND (kidney OR renal OR nephro OR CKD OR ESRF OR ESKD OR transplant OR dialysis OR haemodialysis OR hemodialysis)

**Medline via OVID total: 318**

**IV EMBase (as of 2021.6.30)**

((coronavir OR COVID OR nCoV OR SARS) AND (vaccine OR vaccination OR immunization OR immunisation)) AND (kidney OR renal OR nephro OR CKD OR ESRF OR ESKD OR transplant OR dialysis OR haemodialysis OR hemodialysis)

**EMBase total: 554**
