# Supplementary material for: Immunogenicity and Safety of COVID-19 Vaccines in Patients Receiving Renal Replacement Therapy: A Systematic Review and Meta-Analysis
Source: Front Med (Lausanne). 2022 Mar 9;9:827859. doi: 10.3389/fmed.2022.827859 (PMC8959490; doi:10.3389/fmed.2022.827859)
Supplement: Supplementary file 2 [file Table_2.DOCX]

**S2. Overall risk of bias assessment by the National Institute of Health Study Quality Assessment Tool in included studies**

| **First author** | **Clear objective** | **Clear sampling definition** | **Uniform inclusion and exclusion criteria** | **Sufficient follow-up time** | **Clear definition of vaccination regimen** | **Clear outcomes definition** | **Key confounders measured** | **Quality rating (good, fair, poor)** |
| --- | --- | --- | --- | --- | --- | --- | --- | --- |
| Boyarsky BJ | 1 | 1 | 1 | 1 | 1 | 1 | NR | Good |
| Bertrand D | 1 | 1 | 1 | 1 | 1 | 1 | NR | Good |
| Korth J | 1 | 1 | 1 | 1 | 1 | 1 | NR | Good |
| Benotmane L | 1 | 1 | 1 | 1 | 1 | 1 | NR | Good |
| Danthu C | 1 | 1 | 1 | 1 | 1 | 1 | 1 | Good |
| Rincon-Arevalo H | 1 | 1 | 1 | 1 | 1 | 1 | 1 | Good |
| Rozen-Zvi B | 1 | 1 | 1 | 1 | 1 | 1 | 1 | Good |
| Cucchiari D | 1 | 1 | 1 | 1 | 1 | 1 | 1 | Good |
| Ou MT | 1 | 1 | 1 | 1 | 1 | 1 | NR | Good |
| Marion O | 1 | 1 | 1 | 1 | 1 | 1 | NR | Good |
| Chavarot N | 1 | 1 | 1 | 1 | 1 | 1 | NR | Good |
| Sattler A | 1 | 1 | 1 | 1 | 1 | 1 | NR | Good |
| Marinaki S | 1 | 1 | 1 | 1 | 1 | 1 | NR | Good |
| Grupper A | 1 | 1 | 1 | 1 | 1 | 1 | 1 | Good |
| Grupper A | 1 | 1 | 1 | 1 | 1 | 1 | 1 | Good |
| Anand S | 1 | 1 | 1 | 1 | 1 | 1 | 1 | Good |
| Yanay NB | 1 | 1 | 1 | 1 | 1 | 1 | NR | Good |
| Rodriguez-Espinosa D | 1 | 1 | 1 | 1 | 1 | 1 | NR | Good |
| Lacson E | 1 | 1 | 1 | 1 | 1 | 1 | 1 | Good |
| Agur T | 1 | 1 | 1 | 1 | 1 | 1 | 1 | Good |
| Chan L | 1 | 1 | 1 | 1 | 1 | 1 | 1 | Good |
| Attias P | 1 | 1 | 1 | 1 | 1 | 1 | NR | Good |
| Speer C | 1 | 1 | 1 | 1 | 1 | 1 | 1 | Good |
| Longlune N | 1 | 1 | 1 | 1 | 1 | 1 | 1 | Good |
| Simon B | 1 | 1 | 1 | 1 | 1 | 1 | 1 | Good |
| Jahn M | 1 | 1 | 1 | 1 | 1 | 1 | 1 | Good |
| Broseta-Monzo | 1 | 1 | 1 | 1 | 1 | 1 | 1 | Good |

NR, not reported
