# Supplementary material for: Immunogenicity and Safety of COVID-19 Vaccines in Patients Receiving Renal Replacement Therapy: A Systematic Review and Meta-Analysis
Source: Front Med (Lausanne). 2022 Mar 9;9:827859. doi: 10.3389/fmed.2022.827859 (PMC8959490; doi:10.3389/fmed.2022.827859)
Supplement: Supplementary file 3 [file Table_3.DOCX]

**S3. Assays used for measurement of antibodies in included studies**

| **Assay** | **Cut-off value for seropositivity** | **Number of studies applying the assay** | **Mean antibody titre after 2^nd^ dose in KTR** | **Mean antibody titre after 2^nd^ dose in HD** | **Mean antibody titre after 2^nd^ dose in PD** |
| --- | --- | --- | --- | --- | --- |
| ABBOTT (Abbott Park, Illinois, USA) | ≧50 AU/mL | 7 | 65 ± 233 AU/mL | 1907 ± 2741 AU/mL | - |
| LIAISON (Saluggia, Italy) | ≧13 AU/mL | 5 | 32 ± 77 AU/mL | 467 ± 434 AU/mL | - |
| SIEMENS Healthineers (Erlangen, Germany) | ≧1 IgG index value | 5 | - | 14 ± 15 index value | 16 ± 8 index value |
| Beijing Wantai Biological Pharmacy (Beijing, PR China) | ND | 3 | - | - | - |
| EUROIMMUN (Lübeck, Germany) | >0.8 U/mL | 2 | 1.3 ± 1.1 U/mL | - | - |
| Beckman Coulter Access (Brea California) | ND | 1 | - | - | - |
| ELECSYS (Rotkreuz, Switzerland) | >29 U/mL | 1 | - | - | - |

KTR, kidney transplant recipient; HD, hemodialysis; ND, not documented; PD, peritoneal dialysis.
