# Supplementary material for: Immunogenicity and Safety of COVID-19 Vaccines in Patients Receiving Renal Replacement Therapy: A Systematic Review and Meta-Analysis
Source: Front Med (Lausanne). 2022 Mar 9;9:827859. doi: 10.3389/fmed.2022.827859 (PMC8959490; doi:10.3389/fmed.2022.827859)
Supplement: Supplementary file 4 [file Table_4.DOCX]

**S4. Baseline characteristics of all included studies**

|  | Study first author and country | Number of subjects on RRT | Subjects characteristics | Number of Controls | Vaccine type | Antibody titre assay | Mean timing of blood taking after 2^nd^ dose | AE |
| --- | --- | --- | --- | --- | --- | --- | --- | --- |
| 1 | Boyarsky BJ, et al., USA, 2021 | 322 KTR | - | - | mRNA vaccine unspecified | Anti-S protein (EUROIMMUN) | 29±1 days | - |
| 2 | Bertrand D, et al., France, 2021 | 45 KTR, 10 HD | Mean age 63.5, male 51.1% | - | BNT162b2 | Anti-S protein (ARCHITECT) | 28 days | - |
| 3 | Korth J, et al., Germany, 2021 | 23 KTR | Mean age 57.7, male 48% | 23 | BNT162b2 | Anti-S protein (LIAISON) | 14 days | - |
| 4 | Benotmane I, et al., France, 2021 | 205 KTR | Mean age 57.7, male 64.7% | - | mRNA vaccine unspecified | Anti-S protein (ARCHITECT) | 28 days | - |
| 5 | Danthu C, et al., France, 2021 | 74 KTR, 78 HD | KTR mean age 64.8, male 59.1%; HD mean age 73.5, male 59% | 7 | BNT162b2 | Anti-S protein (LIAISON) | 30 days | - |
| 6 | Rincon-Arevalo H, et al., Germany, 2021 | 40 KTR, 40 HD, 4 PD | Mean age 62.4, male 70% | 35 | BNT162b2 | Anti-S protein (EUROIMMUN) | 7±2days | - |
| 7 | Rozen-Zvi B, et al., Israel, 2021 | 308 KTR | Mean age 57.5, male 64% | - | BNT162b2 | Anti-S protein (ABBOTT) | 21 days | - |
| 8 | Cucchiari D, et al., Spain, 2021 | 117 KTR | Mean age 59, male 70.9% | - | mRNA-1273 | Anti-S protein | 14 days | - |
| 9 | Ou MT, et al., USA, 2021 | 363 KTR | Mean age 60, male 43% | - | BNT162b2 (54%) or mRNA-1273 (46%) | - | - | No severe AE |
| 10 | Marion O, et al., France, 2021 | 121 KTR | Mean age 59, male 63.2% | - | BNT162b2 (99%) or mRNA-1273 (1%) | Anti-S protein (Beijing Wantai Biological Pharmacy Enterprise Co Ltd) | 28 days | No severe AE |
| 11 | Chavarot N, et al., France, 2021 | 101 KTR | Mean age 64 | - | BNT162b2 | Anti-S protein (Beijing Wantai Biological Pharmacy Enterprise Co Ltd) | 30 days | - |
| 12 | Sattler A, et al., Germany, 2021 | 39 KTR, 26 HD | KTR mean age 57.4, male 71.8%; HD mean age 67.4, male 65.4% | 39 | BNT162b2 | - | 8±1 days | - |
| 13 | Marinaki S, et al., Greece, 2021 | 34 KTR | Mean age 60, male 79.4% | 116 | BNT162b2 | Anti-S protein (ABBOTT) | 10 days | - |
| 14 | Grupper A, et al. Israel, 2021 | 136 KTR | KTR mean age 58.6, male 75% | 25 | BNT162b2 | Anti-S protein (LIAISON) | - | - |
| 15 | Grupper A, et al. Israel, 2021 | 56 HD | HD mean age 74, male 75% | 95 | BNT162b2 | Anti-S protein (ABBOTT) | 30±2 days | - |
| 16 | Anand S, et al., USA, 2021 | 1140 HD | - | - | BNT162b2 (62.8%) or mRNA-1273 (34.2%) or J&J/Janssen (3%) | Anti-S protein (SIEMENS) | 30±13 days | - |
| 17 | Yanay NB, et al., Israel, 2021 | 127 HD, 33 PD | Mean age 65, male 63% | 132 | BNT162b2 | Anti-S protein (LIAISON) | - | - |
| 18 | Rodriguez-Espinosa D, et al., Spain, 2021 | 32 PD | Mean age 63.4 | - | mRNA-1273 | Anti-S protein (SIEMENS) | 21 days | - |
| 19 | Lacson E, et al., USA, 2021 | 181 HD, 5 PD | Mean age 67.9, male 52.7% | - | BNT162b2 or mRNA-1273 | Anti-S protein (SIEMENS) | - | - |
| 20 | Agur T, et al., Israel, 2021 | 122 HD, 23 PD | Mean age 71.6, male 66.4% | - | BNT162b2 | Anti-S protein (ABBOTT) | 36±6 days | 2 major AE (1 syncope, 1 pericarditis) |
| 21 | Chan L, et al., USA, 2021 | 41 HD | Mean age 70, male 93% | - | mRNA-1273 | Anti-S protein (ABBOTT) | 7 days | - |
| 22 | Attias P, et al., France, 2021 | 56 HD | Mean age 70 | - | mRNA-1273 | Anti-S protein (Beckman Coulter Access) | 42 days | - |
| 23 | Speer C, et al., Germany, 2021 | 22 HD | Mean age 72.8, male 55% | 46 | BNT162b2 | Anti-S protein (SIEMENS) | - | - |
| 24 | Longlune N, et al., France, 2021 | 85 HD, 24 PD | Mean age 64, male 68.8% | - | BNT162b2 | Anti-S protein (Beijing Wantai Biological Pharmacy Enterprise Co Ltd) | 28 days | No severe AE |
| 25 | Simon B, et al., Austria, 2021 | 81 HD | Mean age 67, male 55% | 80 | BNT162b2 | Anti-S protein (ELECSYS) | 21 days | More local and systemic AE in control group (p<0.0001) |
| 26 | Jahn M, et al., Germany, 2021 | 72 HD | Mean age 68, male 56.9% | 16 | BNT162b2 | Anti-S protein (LIAISON) | 17±2 days | - |
| 27 | Broseta-Monzo, et al., Spain, 2021 | 78 HD | Mean age 67.1, male 67.9% | - | mRNA-1273 | Anti-S protein (SIEMENS) | 14 days | - |

AE, adverse events; anti-S, anti-Spike; KTR, kidney transplant recipient; HD, hemodialysis; mRNA, messenger ribonucleic acid; PD, peritoneal dialysis; RRT, renal replacement therapy
